# Predicting Dihydropyrimidine Dehydrogenase Deficiency and Related 5-Fluorouracil Toxicity: Opportunities and Challenges of *DPYD* Exon Sequencing and the Role of Phenotyping Assays

**DOI:** 10.3390/ijms232213923

**Published:** 2022-11-11

**Authors:** Ottavia De Luca, Gerardo Salerno, Donatella De Bernardini, Maria Simona Torre, Maurizio Simmaco, Luana Lionetto, Giovanna Gentile, Marina Borro

**Affiliations:** 1 Laboratory of Clinical Biochemistry, Advanced Molecular Diagnostic Unit, Sant’Andrea University Hospital, Via di Grottarossa 1035/1039, 00189 Rome, Italy; 2 Department of Neurosciences, Mental Health and Sensory Organs (NESMOS), Sapienza University, Via di Grottarossa 1035/1039, 00189 Rome, Italy

**Keywords:** 5-fluorouracil, *DPYD*, dihydropyrimidine dehydrogenase, next generation sequencing, polymorphism, phenotyping, genotyping

## Abstract

Deficiency of dihydropyrimidine dehydrogenase (DPD), encoded by the *DPYD* gene, is associated with severe toxicity induced by the anti-cancer drug 5-Fluorouracil (5-FU). *DPYD* genotyping of four recommended polymorphisms is widely used to predict toxicity, yet their prediction power is limited. Increasing availability of next generation sequencing (NGS) will allow us to screen rare variants, predicting a larger fraction of DPD deficiencies. Genotype–phenotype correlations were investigated by performing *DPYD* exon sequencing in 94 patients assessed for DPD deficiency by the 5-FU degradation rate (5-FUDR) assay. Association of common variants with 5-FUDR was analyzed with the SNPStats software. Functional interpretation of rare variants was performed by in-silico analysis (using the HSF system and PredictSNP) and literature review. A total of 23 rare variants and 8 common variants were detected. Among common variants, a significant association was found between homozygosity for the rs72728438 (c.1974+75A>G) and decreased 5-FUDR. Haplotype analysis did not detect significant associations with 5-FUDR. Overall, in our sample cohort, NGS exon sequencing allowed us to explain 42.5% of the total DPD deficiencies. NGS sharply improves prediction of DPD deficiencies, yet a broader collection of genotype–phenotype association data is needed to enable the clinical use of sequencing data.

## 1. Introduction

The anti-cancer drugs fluoropyrimidines (FP), including the antimetabolite 5-fluorouracil (5-FU) and its prodrugs tegafur and capecitabine, are widely used to treat solid tumors, mainly colorectal cancers.

Severe toxicity (grade 3–4) including gastrointestinal reactions, myelosuppression, mucositis, nervous system toxicity, and cardiotoxicity, develops in up to 30% of patients and leads to death in about 1% of cases [1,2,3,4,5]. Considering the hundreds of thousands of cancer patients annually treated with FP [4,5], pre-emptive prediction and early recognition of severe toxicity represent key issues to save patients’ lives. The biological mechanism underlying 5-FU toxicity is an impaired drug metabolism due to the deficient activity of the enzyme dihydropyrimidine dehydrogenase (DPD, encoded by the *DPYD* gene), which catabolizes more than 80% of the administered FP to the inactive metabolite fluoro-dihydrouracil (FDHU). DPD deficiency leads to increased 5-FU plasma concentration and has been recognized since the 1980s as a main tract of 5-FU-treated subjects undergoing severe adverse events [6,7,8,9], opening the way to the pre-emptive testing of DPD activity level (e.g., phenotypic assessment) to identify patients with high risk for toxicities. Two main analytical approaches to DPD phenotyping have been developed and successfully employed to improve FP safety: the determination of the uracil/dihydrouracil ratio in plasma, which estimates the DPD activity level by measurement of the endogenous DPD substrate uracil and its metabolite dihydrouracil, and the direct measurement of DPD enzymatic activity in peripheral blood monocular cells, by biochemical assays [10,11,12,13,14]. Unfortunately, such methodologies have limited diffusion in clinical laboratories, since they require peculiar equipment (such as liquid chromatography and mass spectrometry) and are usually based on homemade protocols [10,11,12,13,14].

The alternative approach to phenotyping assays is the *DPYD* genotyping approach: soon after the first description of DPD deficiency, *DPYD* sequencing revealed the presence of gene variations associated with low enzyme activity and/or FP-induced toxicities [15,16,17], setting the stage for the future development of FP pharmacogenetic testing [18,19,20]. By now, hundreds of gene variations, including mutations and single nucleotide polymorphisms (SNPs), have been described in the *DPYD* gene [21]. Among them, few certainly pathogenic variations have been identified and are currently accepted as pharmacogenetic markers for DPD deficiency: the rs3918290 (also known as *2A, c.1905+1G>A, IVS14+1G>A), a splice-site variant causing exon 14 skipping and production of an inactive protein [15,16]; rs55886062 (*13, c.1679T>G), causing the aminoacidic substitution I560S [22,23,24]; rs67376798 (c.2846A>T), causing the aminoacidic substitution D949V [24]; rs75017182 (c.1129-5923C>G), a deep-intronic splice-site variant causing significant loss of DPD activity, which is in near perfect LD with the *DPYD* haplotype HapB3 including three intronic variants (rs56276561, rs6668296, rs115349832); and the synonymous SNP rs56038477 (E412E, c.1236G>A), which is often used as a tag SNP for HapB3 [25,26].

Screening for the mentioned SNPs is recommended by several medicine agencies and international panels of experts, such as the Clinical Pharmacogenetics Implementation Consortium (CPIC) and the Dutch Pharmacogenetics Working Group (DPWG), which also developed specific guidelines for FP dose adjustment in carrier patients [27,28,29,30]. Even if *DPYD* genotyping achieved capillary diffusion in clinical diagnostic labs, it should be kept in mind that the population frequency of the screened SNPs is around 1–2%, whereas DPD deficiency is present in up to 5% of the general population [4,31]. Thus, a significant fraction of DPD deficiencies, caused by different, rare variations, is unpredictable by the current genotyping approach [31,32,33].

Presently, the growing cost-effectiveness of Next Generation Sequencing (NGS) technology and its growing availability in clinical diagnostic labs are enabling the screening of the entire *DPYD* coding region (or the full gene), allowing the detection of additional rare variants (mutations) [34] that may be causative of DPD impairment and 5-FU toxicity. However, to clearly establish the pathogenicity, and thus the clinical utility, of rare variants detected by NGS, novel genotype–phenotype correlations must be described and analyzed.

In this study, we performed *DPYD* exon sequencing (including intron/exon boundaries) in a cohort of 94 subjects who previously underwent DPD phenotyping by a biochemical assay, namely the 5-FU degradation rate (5-FUDR) [12]. DPD deficiencies are defined by 5-FUDR values below the fifth percentile of the values’ distribution in the general population [32,33]. The study cohort was appositely selected to include most of the DPD deficiencies cases (N = 40) identified by previous phenotyping of about 1000 patients [32,33], with the aim to detect specific associations between rare or novel *DPYD* variants and decreased DPD activity.

## 2. Results

The study group included 40 subjects (60% males) with a 5-FUDR ≤ 0.85 ng/mL/10^6^ cells/min, defined as poor metabolism (PM, e.g., DPD deficiency), and 54 subjects (55.5% males) with a 5-FUDR > 0.85 ng/mL/10^6^ cells/min, defined as normal metabolism (NM) [32]. Mean age did not significantly differ between the PM group and the NM group (67.63 ± 11.73 vs. 68.74 ± 12.19, respectively, *p* = 0.65).

**Figure 1:**
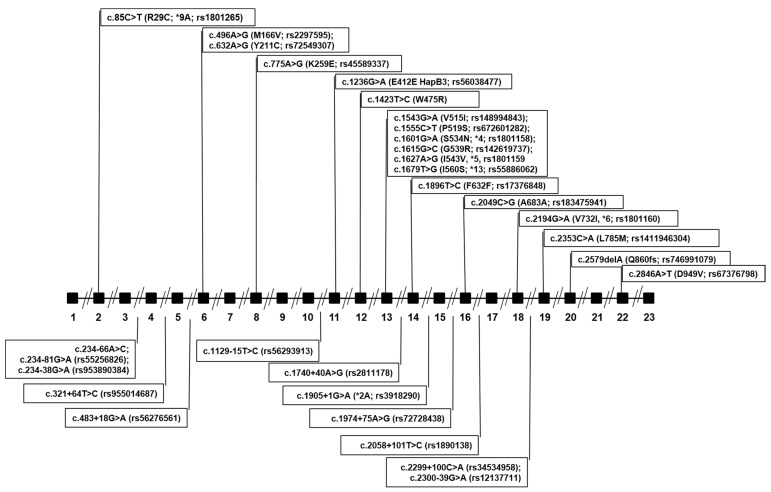
Gene location of *DPYD* variants detected by NGS sequencing. Black boxes represent exons, lines represent introns.

*DPYD* sequencing detected 31 germline variants in the overall population, of which 23 were rare (observed minor allele frequency < 0.05%) and 8 were common (Table 1 and Figure 1). Thirteen variants were present in both PM and NM groups, 11 were detected exclusively in the PM group and 7 exclusively in the NM group. In the PM group, 8 variants were intronic and 16 exonic (13 missense, 2 synonymous and 1 frameshift); in the NM group, 11 variants were intronic and 9 exonic (six missense and three synonymous). A wild-type sequence was found in 3/40 (7.5%) PM subjects and 7/54 (12.96%) NM subjects.

All DNA variations were in Hardy–Weinberg (HW) equilibrium, except the *13 SNP (rs55886062), which was detected only in the PM group and deviated by the HW equilibrium (*p* = 0.038). This result is consistent with the known association of the *13 allele with poor DPD activity [22,23,24].

**Figure 2:**
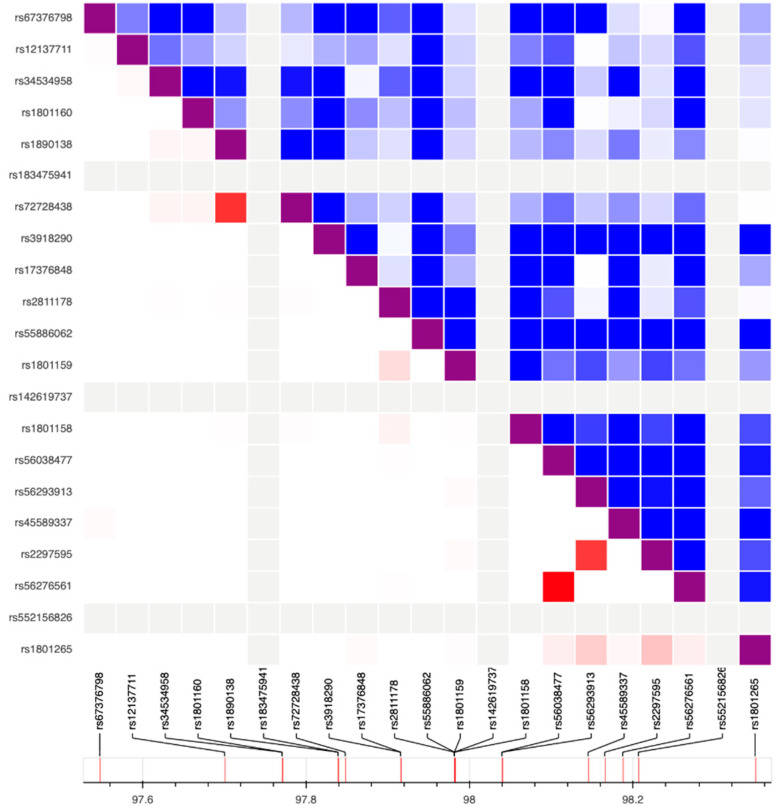
Heatmap matrix of pairwise linkage disequilibrium statistics.

Linkage Disequilibrium (LD) statistics (Figure 2) showed a partial LD between rs72728438 and rs1890138 (D’ = 1.0, r^2^ = 0.795) and between rs2297595 and rs56293913 (D’ = 0.914, r^2^ = 0.762) and a perfect LD between the variants rs56276561 and rs56038477 (D’ = 1.0, r^2^ = 1). The latter association was expected since rs56276561 and rs56038477 belong to the HapB3 haplotype.

**Figure 3:**
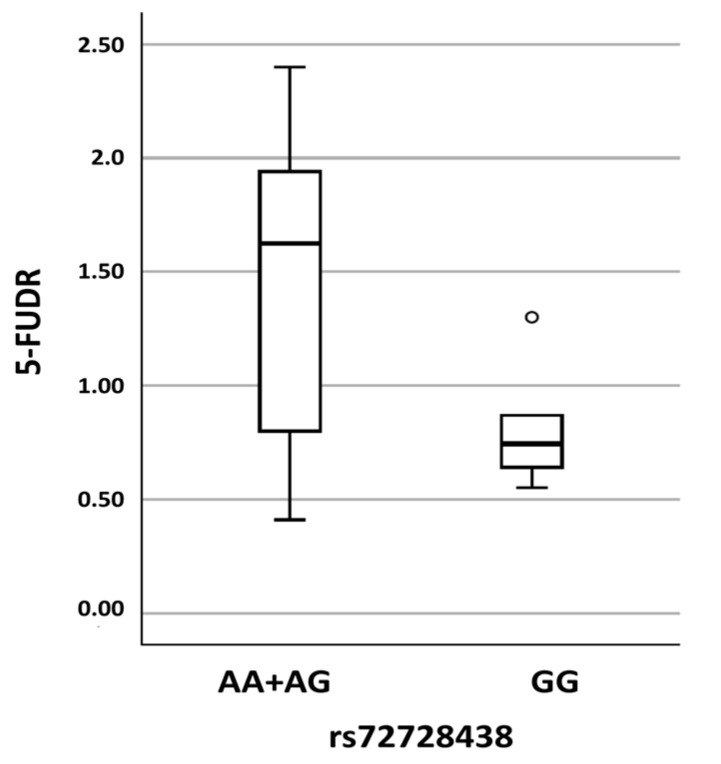
5-FUDR values distribution in subjects with the AA or AG genotype compared to subjects with the GG genotype in the polymorphic site c.1974+75A>G (rs72728438).

Single SNP linear regression analysis testing the seven common polymorphisms (observed minor allele frequency ≥ 0.05), found a significant association between the intronic SNP rs72728438 (c.1974+75A>G) and mean 5-FUDR value (*p* = 0.018) using the recessive models; that is, the mean 5-FUDR was 1.40 ± 0.59 ng/mL/10^6^ cells/min, in subjects with the AA + AG genotype, vs. 0.81 ± 0.26 ng/mL/10^6^ cells/min in subjects with the GG genotype (Figure 3).

Haplotype analysis testing interactions among the seven common SNPs did not detect significant associations with the mean 5-FUDR.

The potential functional effect of rare variants (observed minor allele frequency < 0.05), was investigated in-silico using the Human Splicing Finder System (Genomnis, Marseille, France), to predict the impact of intronic variations on splicing, and PredictSNP [35], to evaluate the impact of missense variations. None of the overall detected intronic variants were predicted to affect splicing, except for c.234-138G>A and c.2300-39G>A, which were predicted to generate an alteration of the exonic splicing enhancer/exonic splicing silencer motifs ratio and to activate a cryptic splicing donor site, respectively. However, both of these variants were detected in NM subjects.

Regarding the missense variants, four were predicted to be deleterious (Y211C, K259E, P519S, G539R) and three non-deleterious (W475R, V515I, L785M). All the missense mutations predicted as deleterious were present only in PM subjects.

## 3. Discussion

The phenotypic 5-FUDR assay was previously established as clinically useful to manage FP treatment. Furthermore, 5% of the general population has 5-FUDR values ≤ 0.85 ng/mL/10^6^ cells/min and is classified as PM [32]. We have previously shown that PM subjects have a significantly increased risk of developing severe 5-FU toxicity, and correlated the presence of known *DPYD* polymorphisms with both 5-FU toxicity and low 5-FUDR values [32,33,36,37,38,39]. Our previous results confirmed that, despite the enormous benefits in terms of treatment safety brought by the system-level genotyping of recommended SNPs, a large fraction of DPD deficiencies remains unpredictable [32,33]. Thus, the implementation of NGS to characterize larger *DPYD* regions is attractive and is becoming more and more actionable. However, broad *DPYD* sequencing will drastically increase the number of reported variants, which will require functional interpretation to be applied to patient therapy management.

In order to highlight novel genotype–phenotype correlations and contribute to the functional assignment of *DPYD* genetic variants, we performed exon sequencing in a patient cohort enriched in DPD-deficient patients (5-FUDR PM group).

Among the eight common variants detected in the overall sample, we found a statistically significant association between the GG genotype in the polymorphic site c.1974+75A>G (rs72728438) and low 5-FUDR (Figure 1). This intronic variant has previously been associated with decreased DPD activity [40], and other studies described its presence in patients with low DPD activity, but the association did not achieve statistical significance [41]. Recently, a study of expression quantitative trait loci (eQTLs), i.e., genetic variants affecting gene transcription and transcript stability [42], found that rs72728443 is in high LD (r^2^ > 0.94) with the intronic *DPYD* variant rs59353118, which is an eQTL significantly associated with reduced *DPYD* expression and with rs12022243 and rs72728443. This latter is located in an enhancer region and spans a p53 binding site. Thus, LD with distant causative polymorphisms may explain the association between intronic rs72728438 and the poor 5-FU metabolism observed in the present and previous reports [40,41].

Confirmation of impaired DPD activity in homozygous carriers of rs72728438 would be of paramount importance; considering that in our sample cohort, the GG genotype was present in 5/40 (12.5%) of PM subjects and no other no-function variants were detected in such subjects, the validation of this marker could drastically improve the genotype-based prediction of DPD deficiency.

Concerning the rare variants identified in this study, the four recommended pharmacogenomic markers can explain 20% of total DPD deficiencies (8/40 PM cases), as follows: *2A (N = 1), *13 (N = 2), HapB3 (N = 4), D949V (N = 1).

Other *DPYD* variations previously reported as deleterious can explain 7.5% of the total DPD deficiencies (3/40 PM cases): c.2579delA (Q860fs, rs746991079), a frameshift variant resulting in protein truncation, previously isolated in individuals with DPD deficiency [43,44]; the Y211C allele, associated with consistent reduction of DPD activity (12.5–25% compared to wild-type) in an in-vitro assay using a recombinant mutant protein [34,45]; and the K259E allele, previously detected in a cohort of 5-FU treated patients undergoing toxicity [34]. One additional PM case (2.5%) could be imputed to the presence of a haplotype (rs1801160, rs1801265, rs2297595) that we previously found to be associated with significantly decreased 5-FUDR [39].

Considering the remaining 57.5% of PM cases, one subject carried the V515I variant, reported as deleterious by Hishinuma et al. [46] (35% DPD activity compared to the wild type) but as functional by Offer et al. (using a different in-vitro assay) [45] and predicted as non-deleterious by in-silico analysis; one subject carried the G539R variant, reported as functional by Offer et al. [45] but predicted as deleterious by in-silico analysis; one subject carried the novel W475R variant (no rsID available); and one subject carried the P519S variant (rs672601282), predicted as non-deleterious and deleterious, respectively, by in-silico analysis. The residual PM subjects had different combinations of known polymorphisms with no effect or uncertain effect on DPD activity or a wild-type sequence (N = 3).

Summing up the above observations, we can roughly compare the common *DPYD* genotyping strategy based on testing a few recommended genetic markers, with the diagnostic scenario opened by the NGS approach. In our sample cohort, pre-emptive genotypic screening limited to the recommended polymorphisms *2A, *13, HapB3 and D949V would have identified just 20% of DPD deficiencies, whereas exon sequencing allowed us to recognize an additional 22.5% of subjects carrying variants, providing a reasonable “warning” for DPD deficiency.

On the other hand, *DPYD* exon sequencing did not reveal a clear genetic determinant for more than a half of the analysed cases of DPD deficiency.

Plainly, sequencing of the full *DPYD* gene will allow us to detect deleterious genetic variations also in regulatory regions. Nevertheless, the concern of sequencing results interpretation should be solved: since most variants detected by sequencing are rare, no clear genotype–phenotype association data are available to support clinical decisions on 5-FU treatment. In-silico prediction and in-vitro expression/activity assays represent good strategies for rapid functional assessment of novel DPD variants, but, as exemplified in our study by the case of the G539R and V515I mutations, functional evaluation from in-silico prediction and in-vitro assay may be discordant, as well as results from in-vitro assays using different systems. Thus, genotype–phenotype association studies remain the main road to produce clinically useful data. It is expected that the increasing adoption of the NGS strategy for *DPYD* screening will expand the collection of data, enabling statistical analysis to recognize strong genotype–phenotype associations. In this scenario, we would highlight that in this type of study, the choice to study a “biochemical DPD phenotype” (e.g., a measure of the patient’s DPD activity level) compared to a “clinical DPD phenotype” (e.g., measure of toxicity following 5-FU treatment) may be preliminarily advantageous. This is because the biochemical DPD phenotype can be measured in the general population despite the presence of cancer, allowing us to drastically increase the number of subjects screened for genotype–phenotype associations. However, we are aware that the clinical validation of a genetic marker identified by such an approach is essential, and that the lack of data about FP-induced toxicity in this study is an objective limit.

## 4. Materials and Methods

### 4.1. Patients

This retrospective study included 94 cancer patients (40 females, 54 males, mean age 68.27 ± 11.95) with a diagnosis of colon cancer (81.9%) or other cancers (18.1%). All patients were tested for 5-FUDR and were categorized as poor metabolizers (5-FUDR ≤ 0.85 ng/mL/10^6^ cells/min, N = 40) or normal metabolizers (5-FUDR > 0.85 ng/mL/10^6^ cells/min, N = 54). The study was conducted according to the Declaration of Helsinki and approved by the Institutional Review Board of Sapienza University (Rif. 3762_2015/23.07.2015, Prot. 2377/2015). Informed consent was obtained from all subjects involved in the study.

### 4.2. 5-FU Degradation Rate

The 5-FUDR assay was determined as previously described [12]. Briefly, peripheral blood mononuclear cells were isolated by Ficoll gradient from EDTA-anticoagulated blood, aliquoted and incubated with a known dose of 5-FU up to 2 h at 37 °C. Cells aliquots were lysed and centrifuged at 0, 1 h and 2 h, then the 5-FU concentration in the supernatants was quantified by HPLC-MS/MS. Furthermore, 5-FUDR is expressed as ng/mL/10^6^ cells/min. Subjects with a 5-FUDR value ≤ 0.85 ng/mL/10^6^ cells/min were categorized as poor 5-FU metabolizers (PMs), whereas subjects with a 5-FUDR value > 0.85 ng/mL/10^6^ cells/min were categorized as normal 5-FU metabolizers (NM).

### 4.3. DPYD Exon Sequencing

Genomic DNA was isolated from 200 mL of EDTA-anticoagulated peripheral blood using the QiaSymphony automatic extractor with the QIAsymphony DSP DNA Mini Kit (Qiagen, Hilden, Germany). *DPYD* target regions including exons and intron/exon boundaries were amplified with an Ion AmpliSeq™ Library Kit 2.0 (ThermoFisher Scientific, Waltham, MA, USA) according to the manufacturer’s instructions. Libraries were then diluted and subjected to templating and chip loading using the Ion Chef™ Instrument with the Ion 510™ and Ion 520™ and Ion 530™ Kit–Chef; NGS was then performed on the Ion S5 System and data were analyzed using the Ion Reporter Software version 5.18 (ThermoFisher Scientific, Waltham, MA, USA).

### 4.4. In-Silico Prediction of Variants’ Effect

Functional consequences of rare genetic variations in the intronic regions (intron/exon boundaries) were analysed using the Human Splicing Finder System (Genomnis, Marseille, France), which evaluated their potential effects on all splicing signals including acceptor and donor sites, branch points and auxiliary splicing signals, such as exonic splicing enhancer/silencer (ESE/ESS).

Functional consequences of rare genetic variations in the exonic regions were analysed using the PredictSNP algorithm, which combines data from different well-established prediction tools to predict the impact of aminoacidic substitutions on protein function [35]. Rare variants were defined as variants with an observed minor allele frequency < 0.05.

### 4.5. Statistics

Numerical variables were expressed as mean ± standard deviation. Tests for deviation from the Hardy–Weinberg (HW) equilibrium, analysis of genotype and allele distributions and association analysis with the 5-FUDR values were performed using the SNPStats online tool [47,48]. Single SNP association with the response variable 5-FUDR was tested using linear regression under a dominant, recessive, co-dominant or log-additive model. Haplotype association with the response variable 5-FUDR was tested using linear regression under a log-additive model. All analyses were adjusted by age and sex. No correction for multiple testing was applied and P values less than 0.05 were considered significant. Single SNP analysis and haplotype analysis were only performed on common variants (N = 7, observed MAF > 0.05, Table 1).

The presence of LD among all variants was evaluated using the web-based application LDlink [49]. The software calculates D prime (D′) and R squared (R^2^) statistics using data from the 1000 Genomes Project [50]. The LD analysis was performed in the European population.

## 5. Conclusions

The current *DPYD* genotyping approach to the identification of patients with high risk to develop severe 5-FU toxicity is limited to the screening of four recommended variants, detecting a minor fraction of actual DPD deficiencies. The advent of cost-effective NGS analysis will allow to detect a high number of rare *DPYD* variants and is expected to greatly improve the prediction power of genetic testing. Though, prerequisites for full implementation of *DPYD* NGS analysis in clinical diagnostics is the collection of further genotype–phenotype association studies to unambiguously define the functional impact of rare variants. In this scenario, we would highlight the key role of DPD phenotyping assays: *DPYD* sequencing in a specific target population identified as “DPD-deficient” by biochemical phenotyping, compared to clinical phenotyping (e.g., response to 5-FU treatment), is simpler and may accelerate “cases” enrollment, increasing the available sample size. Such preliminary identification of novel pharmacogenomics markers would in turn facilitate their clinical validation.

## Figures and Tables

**Table 1:** List of identified *DPYD* variants.

*DPYD* Variants Identified in Both the NM Group and the PM Group							
Locus (hg19)	Nomenclature	dbSNP rsID	Genotype	Frequencies			Notes *
				NM (n, %)	PM (n, %)	Observed MAF	
chr1: 98348885	c.85C>T, R29C, *9A	rs1801265	TT TC CC	30 (56) 19 (35) 5 (9)	22 (55) 15 (38) 3 (8)	0.266	CPIC/DPWG: Fully functional
chr1: 98206101	c.234-66A>C	Not available	AA AC	52 (96) 2 (4)	39 (98) 1 (2)	0.016	In-silico prediction: No consequences on splicing
chr1: 98187048	c.483+18G>A	rs56276561	GG GA	53(98) 1(2)	36 (90) 4 (10)	0.026	In-silico prediction: No consequences on splicing
chr1: 98165091	c.496A>GM166V	rs2297595	AA AG GG	41 (76) 10 (19) 3 (6)	25 (62) 14 (35) 1 (2)	0.170	CPIC/DPWG: normal function
chr1: 98039541	c.1129-15T>C	rs56293913	TT TC CC	39 (72) 13 (24) 2 (4)	26 (65) 13 (32) 1(2)	0.170	In-silico prediction: No consequences on splicing
chr1: 98039419	c.1236G>A, E412E HapB3	rs56038477	GG GA	53 (98) 1 (2)	36(90) 4 (10)	0.026	CPIC/DPWG: reduced function
chr1: 97981421	c.1601G>A, S534N, *4	rs1801158	GG GA	52 (96) 2 (4)	36 (90) 4 (10)	0.032	Insufficient evidence due to contrasting results
chr1: 97981395	c.1627A>G, I543V, *5	rs1801159	AA AG GG	40 (74) 14 (26) 0 (0)	31 (78) 8 (20) 1 (2)	0.127	CPIC/DPWG: fully functional
chr1: 97981242	c.1740+40A>G	rs2811178	AA AG GG	12 (22) 28 (52) 14 (26)	8 (20) 20 (50) 12 (30)	0.468	In-silico prediction: No consequences on splicing
chr1: 97981242–97981243	c.1740+39_1740+40	rs796315813 (MNV)	AC/AC AC/GC GC/GC AC/GT GT/GC	11 (20) 21 (39) 8 (15) 8 (15) 6 (11) 0 (0)	8 (20) 17 (42) 7 (18) 3 (8) 4 (10) 1 (2)	0.271	In-silico prediction: No consequences on splicing
chr1: 97915624	c.1896T>C, F632F	rs17376848	T/T T/C	50 (93) 4 (7)	39 (98) 1 (2)	0.026	Synonymous
chr1: 97847874	c.1974+75A>G, p.?	rs72728438	AA AG GG	33 (61) 20 (37) 1 (2)	20 (50) 15 (38) 5 (12)	0.250	In-silico prediction: No consequences on splicing
chr1: 97770920	c.2194G>A, V732I, *6	rs1801160	GG GA AA	44 (81) 10 (19) 0 (0)	31 (78) 8 (20) 1 (2)	0.106	CPIC/DPWG: insufficient evidence (contrasting results)
***DPYD* Variants Identified Exclusively in the PM Group**							
**Locus hg19**	**Nomenclature**	**dbSNP rsID**	**Genotype**	**Frequencies**			**Notes**
				**NM** **(n, %)**	**PM** **(n, %)**	**Observed MAF**	
chr1: 98206116	c.234-81G>A	rs552156826	G/G G/A	54 (100)	39 (98) 1 (2)	0.005	In-silico prediction: No consequences on splicing
chr1: 98164955	c.632A>G, Y211C	rs72549307	AA AG	54 (100)	39 (98) 1 (2)	0.005	In-silico prediction: Deleterious
chr1: 98144726	c.775A>G, K259E	rs45589337	AA AG	54 (100)	38 (95) 2 (5)	0.011	In-silico prediction: Deleterious
chr1: 98015217	c.1423T>C, W475R	Not available	TT TC	54 (100)	39(98) 1(2)	0.005	In-silico prediction: Non-deleterious
chr1: 97981479	c.1543G>A, V515I	rs148994843	GG GA	54 (100)	38(95) 2(5)	0.011	In-silico prediction: Non-deleterious
chr1: 97981467	c.1555C>T, P519S	rs672601282	CC CT	54 (100)	39 (98) 1 (2)	0.005	In-silico prediction: Deleterious
chr1: 97981407	c.1615G>C, G539R	rs142619737	G/G G/C	54 (100)	39 (98) 1 (2)	0.005	In-silico prediction: Deleterious
chr1: 97981343	c.1679T>G, I560S, *13	rs55886062	TT TG GG	54 (100) 0 (0)	38 (95) 1 (2) 1 (2)	0.016	CPIC/DPWG: no function
chr1: 97915614	c.1905+1G>A, *2A	rs3918290	G/G G/A	54 (100)	39 (98) 1 (2)	0.005	CPIC/DPWG: no function
chr1: 97658667	c.2579delA, Q860fs	rs746991079	A/A A/DEL	54 (100)	39(98) 1(2)	0.005	Frameshift causing stop codon and termination
chr1: 97547947	c.2846A>T, D949V	rs67376798	AA AT	54 (100)	38 (95) 2 (5)	0.011	CPIC/DPWG: reduced function
***DPYD* Variants Identified Exclusively in the NM Group**							
**Locus hg19**	**Nomenclature**	**dbSNP rsID**	**Genotype**	**Frequencies**			**Notes**
				**NM** **(n, %)**	**PM** **(n, %)**	**Observed MAF**	
chr1: 98206173	c.234-138G>A	rs953890384	G/G G/A	53 (98) 1 (2)	40 (100)	0.005	In-silico prediction: alteration of auxiliary splicing sequences
chr1: 98205884	c.321+64T>C	rs955014687	TT TC	53 (98) 1 (2)	40 (100)	0.005	In-silico prediction: No consequences on splicing
chr1: 97839126	c.2049C>G, A683A	rs183475941	G/G G/C	52 (96) 2 (4)	40 (100)	0.011	Synonymous
chr1: 97839016	c.2058+101T>C	rs1890138	T/T T/C	50 (93) 4 (7)	40 (100)	0.021	In-silico prediction: No consequences on splicing
chr1: 97770715	c.2299+100C>A	rs34534958	C/C C/A	53 (98) 1 (2)	40 (100)	0.005	In-silico prediction: No consequences on splicing
chr1: 97700589	c.2300-39G>A	rs12137711	G/G G/A	53 (98) 1 (2)	40 (100)	0.005	In-silico prediction: activation of a cryptic Donor site
chr1: 97700497	c.2353C>A, L785M	rs1411946304	C/C C/A	53 (98) 1 (2)	40 (100)	0.005	In-silico prediction: Non deleterious

MAF: Minor Allele Frequency; MNV: multiple nucleotide variation. * The CPIC/DPWG consensus annotation is reported when available; the other notes report results from in-silico evaluation performed in the present study.

## Data Availability

The authors confirm that the data supporting the findings of this study are available within the article. Raw sequencing data are available from the corresponding author on request.
